# A qualitative exploration of two risk calculators using video-recorded NHS health check consultations

**DOI:** 10.1186/s12875-020-01315-6

**Published:** 2020-12-03

**Authors:** Victoria Riley, Naomi J. Ellis, Lisa Cowap, Sarah Grogan, Elizabeth Cottrell, Diane Crone, Ruth Chambers, David Clark-Carter, Sophia Fedorowicz, Christopher Gidlow

**Affiliations:** 1 Staffordshire University, Brindley Building, Leek Road, Stoke-on-Trent, ST4 2DF UK; 2 Manchester Metropolitan University, Manchester Campus, Bonsall Street, Manchester, M15 6GX UK; 3 Keele University, Keele, Newcastle-under-Lyme, ST5 5BG UK; 4 Cardiff Metropolitan University, Cyncoed Campus, Cyncoed Road, Cardiff, CF23 6XD UK; 5 Stoke-on-Trent Clinical Commissioning Group, Smithfield One Building, Stoke-on-Trent, ST1 4FA UK

**Keywords:** Cardiovascular disease, Risk communication, NHS health check, Chronic disease prevention

## Abstract

**Background:**

The aim of the study was to explore practitioner-patient interactions and patient responses when using QRISK®2 or JBS3 cardiovascular disease (CVD) risk calculators. Data were from video-recorded NHS Health Check (NHSHC) consultations captured as part of the UK RIsk COmmunication (RICO) study; a qualitative study of video-recorded NHSHC consultations from 12 general practices in the West Midlands, UK. Participants were those eligible for NHSHC based on national criteria (40–74 years old, no existing diagnoses for cardiovascular-related conditions, not on statins), and practitioners, who delivered the NHSHC.

**Method:**

NHSHCs were video-recorded. One hundred twenty-eight consultations were transcribed and analysed using deductive thematic analysis and coded using a template based around Protection Motivation Theory.

**Results:**

Key themes used to frame the analysis were Cognitive Appraisal (Threat Appraisal, and Coping Appraisal), and Coping Modes (Adaptive, and Maladaptive). Analysis showed little evidence of CVD risk communication, particularly in consultations using QRISK®2. Practitioners often missed opportunities to check patient understanding and encourage risk- reducing behaviour, regardless of the risk calculator used resulting in practitioner verbal dominance. JBS3 appeared to better promote opportunities to initiate risk-factor discussion, and Heart Age and visual representation of risk were more easily understood and impactful than 10-year percentage risk. However, a lack of effective CVD risk discussion in both risk calculator groups increased the likelihood of a maladaptive coping response.

**Conclusions:**

The analysis demonstrates the importance of effective, shared practitioner-patient discussion to enable adaptive coping responses to CVD risk information, and highlights a need for effective and evidence-based practitioner training.

**Trial registration:**

ISRCTN ISRCTN10443908. Registered 7th February 2017.

**Supplementary Information:**

The online version contains supplementary material available at 10.1186/s12875-020-01315-6.

## Background

Cardiovascular disease (CVD) is the leading cause of death worldwide, accounting for one in four deaths in England [1]. NHS Health Check (NHSHC) is a national programme designed to screen CVD risk, facilitate early diagnosis and reduce health inequalities [2]. All eligible adults, aged 40–74 years, should be invited for NHSHC where CVD risk is assessed based on several risk factors (e.g., blood pressure and cholesterol). Best practice guidance suggests a patient should be given appropriate CVD risk management advice following effective risk communication [3]. However, information on the nature and quality of the consultation is scarce. Insight is limited to patient and practitioner experiences [4], which do not provide a complete understanding of patient-practitioner interactions within the NHSHC.

Communicating risk is challenging [5] and differs according to patient understanding, numerical literacy, and personality traits [6]. Further, emotional responses to risk and the resulting influence on health behaviour varies between patients [7–10]. If delivered sub optimally, risk communication can increase anxiety and reduce confidence in health professionals [11]. Effective risk communication can improve knowledge, empower and create autonomy [12–14]. Within NHSHC, 10-year percentage risk is calculated and communicated to patients using a prediction algorithm, QRISK®2 [with current transference to QRISK®3 [15]], which is populated from new and pre-existing data within the patient’s record. However, most younger eligible adults are predisposed to a lower CVD risk which can lead to false reassurances [16, 17], misinterpretation [18–22], and poor patient recall and confusion [15]. The 2014 JBS risk calculator [15] includes Heart Age [23–26, 27] and 10-year percentage risk, but primarily focuses on lifetime risk of CVD events through CVD event-free survival (Table 1). It also presents information using multiple visual displays (Table 1) [5] and a function to manipulate the scores to show how risk-factor modification affects overall risk (e.g., smoking cessation). Whilst there is some evidence to suggest that lifetime risk, Heart Age and visual displays may be more effective during the communication of risk [25, 26, 28–35], until recently, no research has compared the efficacy of JBS3 and QRISK®2 for communicating risk in NHSHC.

**Table 1:** Features available in each of the risk calculators included in the study

Risk Calculator	Absolute risk (10-year percent-age risk)	Relative risk	Heart Age	CVD Event-Free Survival Age	Icon Arrays	Thermometer scale	Visual Analogue Scale	Bar Chart	Line Graph	Risk Modification Function
QRISK®2	✓	✓	✓		✓					
QRISK®2 + Informatica	✓	✓	✓		✓	✓		✓		✓
JBS3	✓		✓	✓	✓		✓	✓	✓	✓

**Fig. 1:**
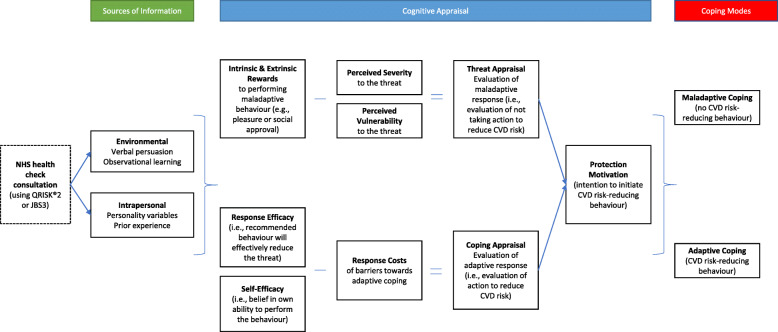
Protection Motivation Theory model adapted to proposed study context [38]

Patient-practitioner interactions are complex [36, 37], yet application of theories such as Protection Motivation Theory (PMT [36];) have shown how fear of threat can translate in to health-protective behaviour [38]. Within PMT, the intention to engage in health-protective behaviour is influenced by an individual’s cognitive appraisals (Fig. 1). CVD risk information presented in an NHSHC can feed into such appraisals, either threat appraisal (risk of CVD), or coping appraisal (consequences of undertaking positive behaviour change).

Threat appraisal focuses on the source of the threat (CVD risk) and evaluates the probability of a maladaptive response (i.e., behaviours that inhibit patients’ ability to adjust to the threat). It considers patients’ perceived severity of CVD risk, the consequences of CVD, perceived vulnerability to future CVD and intrinsic and extrinsic rewards for not addressing CVD risk [i.e., perceived benefits of not acting to manage or reduce risk (maladaptive response)]. Coping appraisal evaluates the adaptive coping responses available to the patient to deal with the threat (i.e., evaluation of ways to reduce CVD risk). This includes patients’ perceptions of self-efficacy to engage in adaptive coping, practitioners’ promotion of self-efficacy through individualisation, perceived response efficacy of adaptive coping, and response cost of adaptive coping (Fig. 1). Both are influenced by intrapersonal (e.g., prior experience of both positive (adaptive) and negative (maladaptive) behaviours) and environmental variables (e.g., persuasive communication) [38]. For NHSHC, PMT highlights the practitioners’ key role in providing information on CVD risk whilst taking into account a patients’ experience, priorities and beliefs to encourage engagement in risk-reducing behaviours [39].

The RIsk COmmunication in NHSHC (RICO) study involved analysis of video-recorded NHSHC consultations [40]. Analysis of quantitatively characterised content of consultations found that compared with JBS3 consultations, those using QRISK®2 were shorter, more verbally dominated by practitioners and involved less discussion of CVD risk [41]. This provided the first insight from objective data on the nature and content of NHSHC consultations, with comparison between risk calculators. But the need for more in-depth qualitative analysis, to explore the quality of interactions around CVD risk and how this differs by CVD risk calculator, was clear. This paper uses deductive thematic analysis on a sample of video-recorded consultations, from the RICO study, which aimed to: explore how practitioners use QRISK®2 and JBS3 to communicate CVD risk in the consultation; explore how patients respond to risk information.

## Methods

### Design

The RICO study sought to explore the perception and understanding of CVD risk from both patients and practitioners, when using the JBS3 or QRISK®2 calculator, the practitioners’ associated advice or offer of treatment and the patients’ response. Information regarding the overall study, including recruitment and data collection is available [40]. In this report, we focus on qualitative deductive analysis of video-recorded NHSHC consultations.

### Participants and recruitment

A detailed description of patient and public engagement along with participant and practice recruitment is available elsewhere [41]. To summarise; data were collected from general practices (*n* = 12) located in the West Midlands of England (Jan-17 to Feb-19), supported by the Clinical Research Network West Midlands. Practices were matched in pairs, based on deprivation, and assigned to usual practice (communicated CVD risk using QRISK®2) or intervention (communicated CVD risk using JBS3). Two practices in the QRISK®2 group used Informatica (supplementary software within in the NHSHC template that includes Heart Age and risk manipulation similar to JBS3; Table 1); data were included in the analysis as this was felt representative of ‘usual care’. Quotations from the transcripts from these practices are referred to as ‘QRISK®2 + Informatica’. Only patients who were eligible for an NHSHC, based on national criteria, were included in the study [42]. Postal invitations included a participant information sheet and were stratified based on gender, age and ethnicity for each practice. Practitioners were already employed by the practice (8 Health Care Assistants (HCAs), 6 Practice Nurses, 1 Sister) and all but one practitioner already had experience of delivering NHSHC as part of their job role (a HCA who was new to NHSHC delivery; 1–2 weeks prior to study commencement).

In total, 175 video-recorded NHSHCs were conducted (range 6.8 to 38 min), reduced to 173 following screening of data (JBS3 = 100; QRISK®2 = 73; practitioner error resulted in 2 exclusions). To define the sample for qualitative analysis, a further 21 Health Checks were excluded for reasons including: projected (not actual) risk score communicated (*n* = 7), no discussion of risk (*n* = 2), no communication of lifetime risk (*n* = 4), incorrect use of JBS3 (*n* = 6), insufficient use of English language (*n* = 2). Of the remaining sample (*n* = 154), 64 Health Checks included communication of CVD risk using QRISK®2. Therefore, 64 NHSHC using JBS3 were identified, matched on patients’ gender, ethnicity and CVD risk score (Table 2), giving a sample of 128 for analysis.

**Table 2:** Characteristics of patients included in analysis

	QRISK®2	JBS3
Gender		
Female	32	32
Male	32	32
Total	64	64
Age		
40–54	34	21
55–64	17	20
65–74	13	23
Total	64	64
Ethnicity		
White British (WBRI)	58	56
Black Asian and Minority Ethnic (BAME)	6	8
Total	64	64
CVD Risk		
Low %	43	43
Med-high %	21	21
Total	64	64

### Procedure

Practices video-recorded NHSHCs, communicating CVD risk using QRISK®2 or JBS3 (following both patient and practice consent). All consultation dialogue was transcribed verbatim.

### Analysis

Data were analysed using deductive thematic analysis [43, 44] using a coding template based around Protection Motivation Theory (PMT [36];) (Supplementary Material 1). Each transcript was uploaded to QSR International’s NVivo 12 qualitative data analysis software [45]. This allowed for interpretation of how QRISK®2 and JBS3 were used to communicate risk in the context of PMT components (e.g., verbal persuasion, influencing patient prior beliefs and priorities; and how patients respond, which will reflect the nature of their appraisal within the consultation).

Initially, 14 transcripts were inductively coded independently by two Caucasian female researchers, experienced in qualitative research, a senior researcher (LC; DPsych) and research associate (VR; MSc). The senior researcher (LC) had previous research experience related to children’s healthy eating whilst the research associate (VR) had previous research experience in risk communication in NHSHC. This was to check the application of PMT to NHSHC consultations and agree coding between the researchers. Following inductive coding, 13 new codes were added to the framework (e.g., medical history, clarification of results). The final version of the coding template shows how elements of the PMT were classified including code definitions and examples from the NHSHC consultations (Supplementary Material 2). The remaining 114 transcripts were individually coded by LC and VR; two in every 20 transcripts were independently dual-coded to check reliability using Kappa coefficients for each NVivo node within the PMT framework (i.e., 19th, 20th, 39th, 40th, 59th, 60th etc). Reliability ranged from .48 to .71 over the five reliability checks conducted, indicating fair to good reliability [46]. Data saturation was considered reached at the point of completion of coding.

Subsequent analysis of codes was led by SF (Researcher; MSc) (supported by SG, CG, NE and VR) to identify codes for key elements of the PMT model, splitting the consultations into two groups (QRISK®2 and JBS3). Specific parts of transcripts that illustrated the practitioner communicating CVD risk to the patient and patient responses were identified. These related to Cognitive Appraisal (Threat Appraisal, and Coping Appraisal), and Coping Modes (Adaptive, and Maladaptive). The focus of the present analysis was the consultation time spent communicating CVD risk (across sample approximately 1.7 (±0.83) minutes) [41], to explore similarities and differences between the two calculators under investigation. Most patients said little in response to CVD risk information. Therefore, where there was evidence of two-way dialogue, we present quotations that best illustrate risk communication and subsequent patient response.

## Results

Deductive thematic analysis was conducted on 128 video-recorded NHSHC consultations. Patients were approximately matched by gender, age and ethnicity. Those in the QRISK®2 group were marginally younger (Table 2).

Results of the deductive thematic analysis demonstrate how practitioners communicated risk using either QRISK®2 or JBS3. They also present patients’ responses to the communication of risk, allowing for evaluation of the two calculators. Each quote is coded to denote which risk calculator was used, the consultation identifier, patient gender and age.

### Cognitive appraisal

#### Threat appraisal

Threat appraisal was the most commonly identified element of the PMT model. It was observed in all consultations, although less frequently in JBS3 consultations (coded 584 times; average 9/consultation) compared to QRISK®2 consultations (coded 634 times; average 10/consultation).

Once presented with a QRISK®2 score, patients acknowledged their risk level, but their understanding of 10-year percentage risk was unclear. For example, one asked ‘*is that percentage of risk alright*?’. Generally, the risk score was acknowledged with a single word response, such as ‘*yeah*’ or ‘*okay*’, impeding practitioners’ ability to gauge patient understanding and classification of response for this analysis. Heart Age aided patient understanding of CVD risk, resulting in questions such as: “*… so really what can I do about that? I mean I know it is all estimated.”* Such questions reflected a level of understanding of the score and intention to engage in risk-reducing behaviour. Several patients expressed surprise at their risk. Below, the patient appeared to question how the score was calculated as they perceived themselves to be healthier than the outcome suggested, leading to some mistrust. They also made two references to being ‘fitter’ than the risk score indicated, which was not addressed by the practitioner:

*P I thought I was fitter than that though.*

*HP (Laughter) You are doing good exercises,*

*P But I was fitter than that though …*

*HP OK, so the health years, so on average expect to survive is 80 for yourself without a heart attack or a stroke, yeah? And then your risk of a heart attack or stroke in the next 10 years is 15%, so you do need to look after yourself, because we would say that is a medium risk.*

*P Yes*

*HP So wouldn’t say it is too high or low, but a medium to high.*

*P OK*

*HP OK, and then that’s what it looks like so from now until there, that’s the last one the chance of surviving without a heart attack.*

*P That’s estimated?*

*HP This is estimated, we don’t know what’s going to happen you might be even longer.*

*P So about 94 I might snuff it?*

* (JBS3, 11_028, Male, 58)*

By overlooking the patient’s surprise and perhaps focusing on the process of NHSHC, the patient momentarily shut down until they were presented with their CVD event-free survival age. The concept, included within JBS3, prompted some misunderstanding among patients and practitioners. This was perceived by some patients as an estimate of life expectancy.

Practitioners provided little follow-up risk score explanation when using QRISK®2 or JBS3.

*HP Right, this is the screening I was telling you about. I will just print that out for you. So your risk of any heart disease is 15%.*

*P Yeah, which is not very high.*

*HP It does increase with age. If it is above 10% we then pass it on for them to have a look at it and they will be able to decide when to have your next health check which should be 3 years or 1 year. Obviously next time you come in any results you’ve got in the red tend to up your risk and they tend to up your Heart Ageas well. So when you come in next time if your blood pressure is back down, and obviously it could be less so … Your Heart Age has come up as 66.*

*P Well I am 66 this year.*

*HP Yes, yes, so it is quite near isn’t it?*

*Yes. So, for example, if you were a smoker and that was in the red that would put your Heart Age at 75. So the only one we have got in the red really is that one cholesterol …*

*P It’s only marginal though isn’t it*

* (QRISK®2 + Informatica, 2_016, Male, 65)*

Above, the patient was identified as medium-high risk, but the practitioner did not elaborate on the severity or implications, leaving the patient’s interpretation of their risk score as “*not very high*”. This was compounded when the patient received their Heart Age. The practitioner did not address the patient’s misinterpretation of the severity of their risk nor explain why their results are conflicting, again perhaps focussing more so on the consultation process than the patient. This led the patient to dismiss their elevated cholesterol as “*only marginal*”. The absence of active listening skills was recurrent across both groups making it difficult to gauge patient understanding.

Although limited, there was more evidence of active practitioner-patient engagement in conversation regarding threat of CVD in the JBS3 group following risk score manipulation (e.g., practitioners visually showed patients that a reduction in blood pressure, could lower their Heart Age):

*HP … so obviously your blood pressure is not too bad, that is fine where it is at 128, but your cholesterol, so ideally we like that to be below 5. So if you could get it below 5, so lets put it down to 4.8, you can see that automatically that it brings your risk down to 1.8%*

*P Oh I see yes*

*HP … improves your life expectancy slightly, and probably brings your Heart Age down a year. So it is just you know showing that it can and obviously, the lower you can keep these factors that you influence, for longer, the better quality of life and life expectancy there is … your risk is going to increase slightly with age. So it is about trying to moderate those other factors.*

*P So what impact does exercise have on that?*

*HP It has quite a significant impact on your cholesterol, it does help your cholesterol a lot. We know that it helps because that increases your good cholesterol, which can help increase the balance so, that can help with it as well.*

*P So what’s the normal range that is seen for HDL cholesterol?*

*HP HDL can be anything from sort of 1.1 to about 2.5, you don’t get much over, I can’t say I have seen many, I have seen a few. But your cholesterol could be anything down to you know 3.5.*

*P OK and really bad would be?*

*HP 6 or 7’s, so would be sort of …*

*P Oh OK – so 5.6 is yeah it is edging up isn’t it?*

* (JBS3, 7_020, Male, 45)*

The patient evaluated the threat and sought information to facilitate their appraisal. Whilst positive, this exchange again demonstrated misunderstanding of CVD event-free survival age as life expectancy, this time from the practitioner. The visual impact of demonstrating how CVD risk can be reduced through risk factor modification (e.g., cholesterol, smoking status) aided patient understanding and realistic threat appraisal. There were fewer examples of active engagement during discussion of the CVD risk score within QRISK®2 consultations, which may be due to the inability to show risk factor modification when using the calculator.

#### Coping appraisal

References to coping appraisal were more common among JBS3 (60, 94%) than QRISK®2 consultations (55, 86%). Communication of risk in JBS3 consultations were not observed in the same way as QRISK2; with most focussed on facilitators of adaptive coping (i.e., risk-reducing changes that patients could make):

*HP Erm and then this gives you your healthy year’s outlook, so based on your current lifestyle your risk of a heart attack or a stroke in the next 10 years is coming out at 2.4%. We aim for peoples risk to be below 10% so that’s …*

*P Yeah.*

*HP … absolutely fine and on average you expected to survive to an age of 84 without a heart attack or stroke, so brilliant. So as I say your blood pressure pretty good as it is you not going get that much lower.*

*P No.*

*HP Diet wise would you say you got a pretty good diet do you know the sorts of …*

*P We sort of grow our own vegetables and fruit and stuff like that …*

*HP Yeah.*

*P … so erm I mean we eat reasonably healthy.*

* (JBS3, 7_044, Female, 54)*

Following communication of the risk score, the practitioner moved on to ways the patient could maintain a low risk through identification of eating behaviours, suggesting that whilst practitioners (from both groups) spent little time talking about the CVD risk score, the additional risk information available in JBS3 may have helped to facilitate more risk factor discussion between the patient and practitioner than when using QRISK®2.

Discussions around response costs for adaptive coping (i.e. perceived costs associated with a recommended behaviour) related to use of statins or blood pressure medication were only observed in seven JBS consultations (11%) and, not any QRISK®2 consultations.

*HP Obviously we’ve tried them, and they haven’t agreed with you.*

P I tried the ***17,34 statin

*HP Yeah, and there are other statins we can discuss and obviously benefits of those they can reduce your cholesterol obviously and we can reduce your risk of cardiovascular disease so it might be worth having a think about and if you want to just discuss that further or a different type of statin …*

*P All they did was it affected my reflux and it made the reflux worse*

*HP Yeah*

*P So*

*HP Yeah*

*P I was on that and an Aspirin – I did the aspirin first and then …*

*HP Yeah, but it was affecting you. I mean it might be worth a having another … err you know a think about whether you wanted to erm take that, because obviously it would lower your cholesterol, obviously add to a healthier heart erm and reduce that risk of cardiovascular disease, but then obviously we’ll not gonna push that onto you, err it is something you can talk to myself, one of the doctor’s once you have had time to think erm and they can advise or XXX the prescribing nurse, because they can prescribe, you know talk about you know what’s best, which statin would be best, and not all statins agree with everybody but there might be one out there that actually has a better erm compatibility with yourself OK?*

*P Yeah*

*HP How do you feel about what I have told you today?*

*P I would consider it.*

* (JBS3, 8_177, Male, 71)*

Here, the patient’s prior engagement with statins as a response cost was discussed between the patient and the practitioner, leading to a re-evaluation of the medical intervention by the patient. However, the patient’s concern regarding their previous experience of taking statins was not well addressed. The practitioner appeared to interrupt the patient to repeat the benefits of statins. The perceived cost of taking statins also provided motivation to adopt risk-promoting behaviours:

*HP But well done!*

*P I am pleased about that yes.*

*HP That’s really good, no I am very pleased with you because that’s really good. And where you were at 10% just before, it is now 5%, so you have halved the risk in that time. So that’s really good. So it shows it can be done.*

*P Yeah, yeah and that’s what I would rather do than taking tablets,*

*HP Of course*

*P I would rather think, no I know what’s wrong, I will deal with it in time.*

* (QRISK®2 + Informatica, 12_055, Female, 64)*

In a previous NHSHC (conducted 5 years prior), the patient identified what was wrong and showed accountability for making health-related behavioural changes, “*I will deal with it*”. However, opportunities to discuss facilitators of adaptive coping were sometimes missed by practitioners:

*HP I look at your [total: HDL cholesterol] ratio and your ratio is good. But just to keep a little eye on it, maybe they will test it again in a year’s time. You probably won’t be due this Health Check, because your risk is only 3%, which is low. It will increase as you age, so your Health Check wouldn’t be due again for 5 years, but you could probably have your cholesterol done in about a year, with you know normal bloods taken out of your arm. Erm your Heart Age, because you got such results in the green, your Heart Age has come up less than your actual age, but that’s with the 2 years added on from being an ex-smoker.*

*P  So is it possible that I could get that even lower, if my cholesterol came down a lot.*

*HP Well we will have a look now, I will play about with it. So if you had never smoked at all, your Heart Age would be 45. If you were still smoking, it could be 51. So being an ex-smoker tends to add 2 years, so with your cholesterol, it could be brought down to 46.*

*P Massively yeah.*

* (QRISK®2 + Informatica, 2_077, Male, 48)*

The patient above attempted to understand how their risk could be reduced. The practitioner did not engage with this to encourage the risk-reducing behaviour or discuss ways to reduce cholesterol. Rather, they proceeded to talk about the impact of previous smoking status (which is unmodifiable) on CVD risk. Whilst references to coping appraisal were more common among JBS3 consultations, again practitioners in both groups appeared to focus more on the consultation process than the patient.

### Coping modes

#### Maladaptive coping

Maladaptive coping was classified when the patient appeared to negatively engage in risk management discussion with the practitioner and was dismissive of suggestions (e.g., patient believes they have a sufficiently healthy lifestyle and dismisses discussion about change). As noted, patient responses to risk information were often limited to single words. Where context allowed, apparent non-engagement and minimal verbal responses from patients were also interpreted as maladaptive coping responses when the risk information communicated by the practitioner did not provoke a response from the patient (i.e., a monosyllabic response). Maladaptive coping was identified in 49 (77%) QRISK®2 consultations (coded 139 times; average 3/consultation), compared to 40 (62.5%) JBS3 consultations (coded 110 times; average 3/per consultation). Below, the practitioner briefly communicates QRISK®2 before moving on to Heart Age (using Informatica):

*HP Yeah this is the screening I was telling you about. So, your risk is 9%*

*P Right*

*HP Which is your key risk for you over the heart disease and diabetes and stroke risk*

*P And heart disease*

*HP As you, as you age your risk does seem to increase, erm any results that you’ve got in the red tend to push up your Heart Age slightly*

*P Aha*

*HP So if we can get the results out of the red and back into the green, that can reduce that one down*

*P Right OK*

*HP So for example, being an ex-smoker actually puts 2 years onto your Heart Age there.*

*P Yeah*

*HP So would be its 66 and it would be 66 if you never smoked at all.*

*P Right.*

*HP Erm if you were still smoking it would be 73.*

*P Oh my gosh*

*HP Your Heart Age has come up as 71 – you are 69. Any results you have got in the red do tend to increase your Heart Age. It is just that one cholesterol one that was in the red.*

* (QRISK®2 + Informatica, 2_001, Female, 66)*

Sometimes maladaptive responses to the 10-year percentage risk score could be prompted into a more positive response through communication of Heart Age. The brief exchange prior to the communication of Heart Age may have also suggested that the practitioner was less confident in discussing absolute risk, a recurrent observation. If practitioners cannot clearly explain the meaning of a patient’s percentage risk score to confer understanding, subsequent discussion/actions regarding risk management may be undermined.

Minimal engagement following communication of the risk score was also identified in JBS3 consultations:

*HP OK. And your blood pressure being under 82 but that’s fine everything is OK with that. Now, on average what they’re saying is that your risk of a heart attack or stroke in the next 10 years is 15%, again, that is down to the fact that you smoke.*

*P Hm*

*HP OK.*

*P Sigh*

*HP And to expect to survive till the age of 78 without a heart attack or a stroke OK. And if we have a look at the next, this one, just reiterates its this, but if I changed it to … say if you didn’t smoke OK and we went to the next your Heart Age would then become equal with your age.*

*P Hm hm*

*HP And your risks in … of a heart attack or stroke in the next 10 years comes down to 9.6% and your actual survival to the age of 83 without a heart attack or a stroke OK and that reiterates it in that as well.*

*P Hm hm*

*HP OK so that’s the difference.*

*P Hm hm*

*P Hm hm*

*HP OK. Erm*

*P Cough*

*HP So it gives you food for thought.*

*P Hmm hmm. You haven’t told me anything I didn’t already know.*

* (JBS3, 1_181, Male, 65)*

The practitioner did not encourage the patient to quit smoking nor did they explore any experience with previous attempts and therefore were unlikely to promote intention to change behaviour. With an added pressure of time within NHSHC consultations, adherence to the process of completing the NHSHC may result in patients being passive recipients of information. As shown above (and throughout), the practitioner delivered the information presented on the screen without asking questions to check understanding or provide context. This resulted in little response from the patient which may be indicative of deference to the practitioner’s health knowledge and is, again, evidence of power imbalance.

Negative engagement in discussion of risk factor management was also evident following the suggestion of statin use:

*HP What we do tend to say if you risk is above 10%, obviously I don’t know whether the doctors have ever discussed a statin with you?*

*P I don’t see the point, I mean if I am going to live to 83, I am quite happy to live to 83.*

*HP So it’s just about being aware that we know that taking a statin can help reduce your overall risk, so it’s one that sort of we usually advise that …*

*P If we do this next time and I don’t know, it was 04 [last cholesterol check], and we are now in 2018, so what does that mean, it could be another 12 to 14 years [for the next Health Check]?*

*HP Well I do normally try and do these every 5 years, so yeah.*

*P So yes, if it is hugely worse*

*HP Yeah*

*P … in 5 years, I will consider it.*

* (JBS3, 7_012, Male, 70)*

Again, the patient misinterpreted CVD event-free survival age and suggested that their risk was not severe enough to consider medical intervention in the short-term; only if it was “*hugely worse*” in the next NHSHC. This was another example of a missed opportunity for the practitioner to question the patient’s understanding of their risk and potential false reassurance provided by the 10-year percentage risk score.

#### Adaptive coping

Adaptive coping was classified when the patient appeared to positively engage with discussion of interventions to manage CVD risk; apparently listened to and engaged in the consultation and accepted what was being said/suggested. Adaptive coping was identified in 58 QRISK®2 (91%) consultations with (310 codes; average 5/consultation) and 55 JBS3 (86%) consultations (328 codes; average 6/consultation). The frequency of occurrences overall and per consultation were similar between the two groups for adaptive coping in medical interventions [39 QRISK®2 (61%) consultations and 116 codes (average 3/consultation); 42 JBS3 (66%) consultations and 142 codes (average 3/consultation)] and lifestyle changes [11 QRISK®2 (17%) consultations and 15 codes (average 1/consultation); 20 JBS3 (31%) consultations and 32 codes (average 2/consultation)]. A number of patients showed intentions to change behaviour as a result of their CVD risk.

*HP So your ratio is 3.5. So this is the screening I was telling you about. So your risk is 3%. That will increase as you age.*

*P Yeah*

*HP And obviously if we can, perhaps with your smoking, it has pushed your Heart Age up to 48, and your age is 41. Because that is the only result you have got in the red. Because all your other results are really good, they are in the green.*

*P They are really good, so I need to …*

*HP Yeah, so if you had never smoked at all, your Heart Age would be aged 40.*

*P I think I need to do something about that don’t I?*

* (QRISK®2 + Informatica, 2_122, Male, 41)*

Here is another example of how Heart Age changed the way the patient responded to the information presented. Whilst a positive response was received, little time was allowed to respond before the practitioner moved on. Giving time for the patient to check their understanding with the practitioner may have provided opportunity for the patient to increase their confidence in actively engaging with coping behaviours. Another example of positive engagement during the discussion of risk was also identified in another practice:

*HP OK that’s good. Err let’s see your key risk.*

*P If I know what weight so I can just try to change my life.*

*HP Yeah, yeah it would be good if you can cut down and, and lose a bit of the weight err what was it 13.8. So it’s only a little higher it should ideally be below 10% is what we want so 13.8 is a bit high but it is because of, because of your weight. OK you don’t smoke you don’t drink alcohol so that’ all good, but your waist is a bit big as well.*

*P Yeah*

*HP Your waist is erm it’s 112 let’s have a look.*

*P Around my tummy around here.*

*HP Yeah let’s have a look. So your waist is 44 in..*

*P And that’s this bit here.*

* (QRISK®2, 3_259, Male, 57)*

The patient above engaged in the information presented about their risk and suggested a need for weight management, somewhat reinforced by the practitioner. However, the interaction was disjointed, which may be a result of the practitioner’s need to complete all elements of the NHSHC and attending to what the patient is saying, creating a barrier for adaptive coping. Whilst scarce, a successful strategy for supporting adaptive coping used by one practitioner was to ask the patient to reflect on the risk information they had received, prompting consideration of action needed:

*HP So average survival free of heart attack or stroke is 84.1 years OK? So how do you feel about that?*

*P Oh I will make more of an effort to lose some weight.*

* (JBS3, 1_154, Female, 70)*

The approach adopted by the practitioner encouraged the patient to express their immediate reaction to their CVD risk, which gave the patient time to evaluate their action and show intention to change their behaviour. This was a rare example of the PMT in action; showing connection between risk information and the patient’s intention to change her behaviour, helping to redress the power imbalance evident in most consultations across both groups. It also demonstrated the significant role the practitioner plays in ensuring risk communication is delivered effectively regardless of the risk calculator used.

## Discussion

We report the first qualitative data from 128 video-recorded NHSHCs to explore how practitioners use QRISK®2 and JBS3 to communicate CVD risk in the consultation, and how patients respond to risk information. An ecologically valid approach was used to compare usual practice (QRISK2) with use of JBS3 following basic introductory training to familiarise practitioners with the tool and features to use. This allowed a realistic study of how practitioners would use JBS3 if it was made available, without additional risk communication training, which is generally not provided for NHSHC practitioners [4, 47].

Main findings in relation to our aims were, first, that components of the PMT including threat appraisal, facilitators of and response costs to adaptive coping were coded more frequently in consultations using JBS3 (compared with QRISK2). This suggests that JBS3 may provide more opportunities to initiate risk factor discussion than QRISK2, possibly due to the risk factor modification function. Second, CVD event-free survival age communicated in JBS3, was misunderstood by both patients and practitioners. Third, patients presented with a QRISK®2 score acknowledged their risk level, but it was unclear whether they understood 10-year percentage risk (or trusted the basis and relevance to them). Visual presentations of risk and Heart age, found in JBS3 (not typically communicated within standard practice systems - although can be generated in QRISK®2), appeared more impactful and aided patient understanding, compared with QRISK®2. This is in line with evidence that Heart Age is easier to understand than 10-year percentage risk [29, 48] and visual displays are preferable for promoting risk-reducing behaviour [30].

Regardless of the risk calculator used and despite the recognised importance of risk communication in both the NHSHC best practice guidance [3] and competence framework [49], there was little discussion of CVD risk. This was particularly marked in QRISK®2 consultations. Practitioners often simply relayed the risk score, without discussing the implications of the risk for the patient or what they could do about it. Equally, most patients offered minimal responses to the risk information, often acknowledging with a single word. Practitioners may have avoided confirming patient understanding if they felt unable to explain the risk scores in more detail or the pressure of time may have prevented further exploration at the expense of the quality of risk communication. This supports evidence that patients and practitioners struggle to understand CVD risk and some practitioners lack confidence in communicating the risk score [4, 17, 19–21] leading to poor patient recall of CVD risk, confusion [22] and misunderstanding.

There was an apparent absence of active listening by practitioners who frequently missed cues from patients who were unclear about their risk score. Active listening involves making a conscious effort to focus on what is being said rather than passively ‘hearing’ the message, and leads to improved levels of patient satisfaction and greater adherence to treatment options [50]. By not providing additional information to patients that would allow them to appraise their risk, practitioners are limiting the opportunity for patients to show intent to engage in risk reducing behaviours, thus encouraging a maladaptive coping response. Best practice guidance [3] recommends that practitioners use motivational interviewing (MI) to encourage adherence to recommended treatment [51]. Motivational interviewing is a person-centred approach to promote discussion with patients to resolve ambivalence [52]. There was little to no evidence of MI techniques in our 128 NHSHC consultations.

Limited patient responses and poor listening skills, leading to practitioner dominance, were inferred from quantitative analysis of the complete RICO study cohort [*n* = 173 [41]]. These were confirmed here, with evidence of missed opportunities to discuss patients’ intentions to change behaviour. Missing these opportunities risks undermining the purpose of the NHSHC; without discussion of intervention practitioners are unlikely to encourage patients to commit to engaging in risk-reducing behaviours. The demands on practitioners to complete all aspects of an NHSHC within a limited time could lead to prioritisation of process over patient engagement. The resulting practitioner-dominated consultations are less patient-centred, and would be expected to lead to low patient and practitioner satisfaction [53–56], and poor patient outcomes, such as adherence to clinical recommendations and health-promoting behaviour [57]. Where there was talk of risk-reducing behaviour, JBS3 appeared more effective than QRISK®2 in promoting discussion of facilitators for adaptive coping, perhaps due to additional functionality (i.e., manipulation of risk). This suggests that other methods of communicating risk may be more suitable to promote discussion around risk-reducing behaviour.

### Implications for practice

The NHSHC programme is an ambitious non-communicable disease prevention programme, the largest of its kind [4]. An evidence-based review of NHSHC is underway to maximise the programme’s benefit in the next decade, with likely changes to the universal offer of in-person consultations in primary care [58]. Whilst changes to delivery are inevitable, elements of the programme will still require practitioner-patient consultation. Our findings show that certain functions of JBS3 are useful for communicating CVD risk to patients, and also highlighted important implications for NHSHC practice in general:

- There is a clear training need among NHSHC practitioners. There is an expectation that practitioners ‘should be trained in communicating the risk score and results to the client’ and that ‘methods, such as motivational interviewing techniques, should engage clients in person-centred conversations about their own reasons for change’ [49] (p21). Yet it is difficult for practitioners to meet these requirements without necessary training and ongoing support. Here, these skills were generally not evident and we know from previous work that practitioners responsible for delivering NHSHC generally receive little (or no) training in CVD risk communication and motivational interviewing [22, 59, 60].
- Alongside training, there is also a need to prioritise the quality of interaction over the process of the consultation. Increasing the overall appointment length or, perhaps more feasible, streamlining the components of NHSHC would give practitioners more time to engage patients in dialogue regarding their CVD risk and its management. The minimal response from patients during the NHSHC consultations made it difficult for us (and practitioners) to gauge patient understanding and intentions for health-promoting behaviour.

Positive outcomes were identified when practitioners checked patient understanding, relayed information in a way that was meaningful to the patient (e.g. Heart age) and asked for patient feedback around the CVD risk score. Practices included in the sample allocated 15–30 min per Health Check, but our quantitative evidence showed consultations lasted as little as 6.8 min [41]. There is clearly a need to provide additional support for practitioners. Measures to make consultations more patient-focused and give practitioners the flexibility to allow engagement in dialogue should be explored.

### Strengths and limitations

This is the first qualitative analysis to explore how risk is communicated and how patients respond during video-recorded NHSHC consultations, including comparison of QRISK®2 and JBS3 CVD risk calculators. Strengths include video-recording of NHSHCs across a diverse range of practices stratified by deprivation, with stratified sampling of patients, a comprehensive coding approach and a large sample (for qualitative analysis). Limitations are recognised:

- The use of QRISK®2 *+ Informatica* may have enhanced these consultations. To maintain ecological validity of ‘usual practice’, patients from these practices were included in the main analysis and has not altered our conclusions.
- Incorrect use of JBS3 (e.g., including communication of CVD event-free survival) resulted in the exclusion of several consultations which may have biased our comparisons in favour of JBS3.
- Sparse discussion specifically around the risk score and subsequent patient responses made it difficult to apply the PMT framework effectively (the theoretical framework required researchers to classify patient responses as either positive or negative). Thus, a third ‘neutral’ classification was added to the framework to account for monosyllabic responses (see Supplementary Material 2 for examples). Yet following the PMT, the new category still needed to be classified as one of the two coping modes (i.e., adaptive or maladaptive). Moreover, follow-up interviews with patients and practitioners as part of the RICO study, will be analysed to further explore their experiences, perceptions and understanding of CVD risk and related intentions.

## Conclusions

Analysis of video-recorded NHSHC consultations showed sparse communication of CVD risk, particularly in consultations supported by QRISK®2. Where risk was communicated, patient responses were minimal and practitioners missed opportunities to check patient understanding and encourage risk-reducing behaviour. JBS3 appeared to better promote opportunities to initiate risk-factor discussion and Heart Age and visual representation of risk were more easily understood and impactful than QRISK®2. The apparent lack of effective CVD risk discussion in both groups resulted in misunderstandings, practitioner-dominated discussion and increased likelihood of a maladaptive coping response. The NHSHC programme is currently the largest CVD prevention initiative in England. Whilst an evidence-based review of NHSHC is underway [52], with likely changes to programme delivery, face to face consultations are necessary to deliver key elements of NHSHC. The analysis presented demonstrates the importance of effective, shared practitioner-patient discussion for enabling adaptive coping responses, only achievable through solid practitioner understanding of the nature of the information being shared and through effective training to deliver this information to patients [60].

## Supplementary Information

**Additional file 1: Supplementary Material 1.** Original PMT Deductive Coding Table.

**Additional file 2: Supplementary Material 2.** Final PMT Deductive Coding Table (following inductive coding including examples).

## Data Availability

All data generated and analysed during the current study are not publicly available due to the confidential nature of participant transcript data, but are available from the corresponding author on reasonable request.

## Abbreviations

CVD: Cardiovascular Diseases
NHS: National Health Service
NHSHC: National Health Service Health Check
RICO: RIsk COmmunication Study
PMT: Protection Motivation Theory
JBS: Joint British Societies
HCA: Health Care Assistant
