# Supplementary material for: A qualitative exploration of two risk calculators using video-recorded NHS health check consultations
Source: BMC Fam Pract. 2020 Dec 3;21:250. doi: 10.1186/s12875-020-01315-6 (PMC7716424; doi:10.1186/s12875-020-01315-6)
Supplement: Supplementary file 1 — Additional file 1: Supplementary Material 1. Original PMT Deductive Coding Table. [file 12875_2020_1315_MOESM1_ESM.docx]

**Supplementary Material 1 - PMT Deductive Coding Table**

|  |  | **Example codes** |
| --- | --- | --- |
| **Sources of Information** | ***Environmental*** | - Family history of CVD - Experience of family members with CVD (+ve/-ve/neutral) |
|  | ***Intrapersonal – Personality Variables*** | Biomedical Risk Factors   - Blood pressure - Cholesterol level - Diabetes - Weight & BMI - Waist circumference - Age - Gender   Lifestyle Behavioural Factors   - Physical activity - Diet - Smoking status - Alcohol consumption - Taking of legal/illegal drugs   Psychosocial Risk Factors   - Social support - Social isolation - Optimism - ‘Felt age’ - Health locus of control/fatalism   Mental Health and Well-being   - Stress - Distress - Anxiety/depression |
|  | ***Intrapersonal – Prior Experience*** | - Prior experience of CVD risk reduction (+ve/-ve/neutral experience)   - Use of statins   - Smoking cessation   - Attempts to reduce weight   - Attempts to undertake physical activity   - Attempts to modify diet |
| **Cognitive Appraisal** | ***Threat Appraisal*** | Discussion of Risk   - Perceived severity of CVD risk (high/low/neutral level of severity) - Consequences of CVD - Perceived vulnerability to future CVD/CVD-related events (high/low/neutral personal relevance) - Intrinsic and extrinsic rewards for not addressing CVD risk |
|  | ***Coping Appraisal*** | Discussion of Risk   - Self-efficacy to engage in adaptive coping   - Promotion of self-efficacy through individualisation - Response efficacy of adaptive coping - Response cost of adaptive coping - Biomedical intervention/lifestyle intervention subcategories for each category & +ve/-ve/neutral subcategories |
| **Coping Modes** | ***Adaptive Coping*** | Biomedical Intervention   - GP appointment referral - Medications (for blood pressure/lipids)   Lifestyle Intervention   - Referral to lifestyle programmes? - Weight management - Increase physical activity - Diet related   - Increase fruit and vegetable intake   - Increase omega-3 fatty acid intake (e.g., fish)   - Decrease fat intake   - Decrease dietary cholesterol   - Decrease sugar intake   - Decrease alcohol intake   - Decrease salt intake - Smoking cessation   Psychosocial Intervention  Positively engaged with biomedical/lifestyle/psychosocial intervention discussion - apparently listening and engaged in the conversation; accepting of what is being said/suggested. |
|  | ***Maladaptive Coping*** | Negatively engaged with biomedical/lifestyle/psychosocial intervention discussion - apparently listening and engaged in the conversation, but dismissive of what is being said/suggested (already doing all I can (e.g., already feels that they are very active and eat well); not interested in making changes suggested (e.g., like smoking; hate physical activity)  Passive/disengaged with biomedical/lifestyle/psychosocial intervention discussion – not engaged in the conversation (e.g., passively takes information, but no clear plans for further contact). |
