# Supplementary material for: A qualitative exploration of two risk calculators using video-recorded NHS health check consultations
Source: BMC Fam Pract. 2020 Dec 3;21:250. doi: 10.1186/s12875-020-01315-6 (PMC7716424; doi:10.1186/s12875-020-01315-6)
Supplement: Supplementary file 2 — Additional file 2: Supplementary Material 2. Final PMT Deductive Coding Table (following inductive coding including examples). [file 12875_2020_1315_MOESM2_ESM.docx]

**Supplementary Material 2 - PMT Deductive Coding Table (final version including examples)**

|  |  | **Codes** | **Example Quote (if available)** |
| --- | --- | --- | --- |
| **Sources of Information** | ***Environmental*** | - Family history of CVD and/or CVD-related factors | **HP:** Is there any family history, is there mum, dad, any brothers or sisters that were diagnosed with any form of blood pressure problems, strokes, heart problems, under the age of 60?  **P:** No. Mum and dad are both 88, still living. They are now on heart sort of tablets and things though. |
|  |  | - Experience of family members with CVD and/or CVD-related factors (+ve/-ve/neutral) | *Positive Experience:*  No example available.  *Neutral Experience:*  **P:** That’s alright. My Dad did suffer from high BP.  *Negative Experience:*  No example available. |
|  |  | - Experience of GP   *The patient discusses motivating or demotivating factors for attending the Health Check in relation to their experiences of going to the GP and how they usually approach healthcare.* | **HP:** So have you ever had your cholesterol and that done?  **P:** No I’ve never no I’m one of the ones that tries to avoid the doctors at all costs so…    **HP:** Thanks for coming in  **P:** Oh no, I always say yes to these things (laughter) |
|  |  | - Environmental influences   *External influences that may have an impact on CVD risk factors (e.g., impact of work on exercise levels).* | **P:** Well I’m ex-army so a big part of my …  **HP:** Yeah so you are used to it.  **P:** I joined the army when I was 15, so a big part of my life was in the army.  **HP:** Ah oh yeah, the marching yeah. |
|  |  | - Influence of significant others   *This category relates to discussion surrounding the positive or negative effect that significant others may have on their CVD risk factors or adaptive/maladaptive coping.* | **HP:** And its that’s how you know how you feed your farmers up isn’t it?  **P:** But you know he’s out in horrific weather, he needs good food. |
|  | ***Intrapersonal***  ***Personality Variables*** | Biomedical Risk Factors   - Blood pressure - Cholesterol level - Diabetes - Weight & BMI - Waist circumference - Age - Gender - Urine - Pulse - Height | Evidence related to discussion of biomedical CVD risk factors. |
|  |  | Lifestyle/Behavioural Factors   - Physical activity - Diet - Smoking status - Alcohol consumption - Taking of legal/illegal drugs | Evidence related to discussion of lifestyle/behavioural CVD risk factors. |
|  |  | Psychosocial Risk Factors   - Social support - Social isolation - Optimism - ‘Felt age’   - Feeling younger/older than chronological age. - Health locus of control/fatalism   - The belief that health outcomes are contingent upon (a) personal behavior (Internal HLC), (b) other powerful people (Powerful Others HLC), and (c) forces such as fate or chance (Chance HLC). | Evidence related to discussion of psychosocial CVD risk factors. |
|  |  | Mental Health and Well-being   - Stress - Distress - Anxiety/depression | Evidence related to discussion of mental health and well-being CVD risk factors. |
|  |  | - Medical history   *Patient discusses intrapersonal variables relating to their medical history (e.g., previous blood pressure measurements, previous weight measurements etc.) This information would also be coded under the relevant medical risk factor.* | **HP**: Have you ever received treatment for blood pressure problems?  **P**: Low blood pressure, not treatment for it, I have had a test for it, because it was very low at one time, but I think it goes up and down. I have just run a marathon not so long ago, so that might have had an effect.  **P:** Yeah, I’m due to have another one… I didn’t have cholesterol done I just had sugar  **HP:** Your cholesterol’s in date actually it’s come up as blue on here  **P:** Oh has it what does that mean  **HP:** Which means it was done last year, it was done in August last year. |
|  | ***Intrapersonal***  ***Prior Experience*** | - Prior experience of CVD risk reduction (+ve/-ve/neutral experience)   - Use of statins   - Smoking cessation   - Attempts to reduce or maintain weight   - Attempts to undertake physical activity   - Attempts to modify or maintain diet   - Attempts to reduce or monitor alcohol consumption   - Attempts to reduce blood pressure   - Attempts to make or maintain lifestyle changes | *Positive Experience (smoking cessation)*  **P:** And the only you know, and in fact I saw the Help to Quit nurse about 10 years ago when I stopped erm I mean she was a very, very unhealthy specimen she was an enormous obese lady.  **HP:** Oh right.  **P:** But she was very good, very good.  *Negative Experience (modifying/maintaining diet)*  **P:** Slimming World I could never accept.  *Neutral Experience (undertaking physical activity)*  **HP:** Yeah, so do you ever manage to go to the gym, or swimming, or any exercise classes?  **P:** I used to go for the gym |
|  | ***Current Experience*** | - Current experience of CVD risk reduction (+ve/-ve/neutral experience)   - Use of statins   - Smoking cessation   - Attempts to reduce or maintain weight   - Attempts to undertake physical activity   - Attempts to modify or maintain diet   - Attempts to reduce or monitor alcohol consumption   - Attempts to reduce blood pressure   - Attempts to make or maintain lifestyle changes | *Positive Experience (alcohol consumption)*    **P:** Some people just pour it in, don’t they?  **HP:** Aye they do, they do.  **P:** I am aware, and if I think I have perhaps have had more than I should in the week, then I stop and don’t drink at all.  *Negative Experience (modifying/maintaining diet)*  No example available.  *Neutral Experience (modifying/maintaining diet)*  **P:** I think I probably eat more meat than veg.  **HP:** Right  **P:** If in the whole scheme of things.  **HP:** Yeah  **P:** Which is something I’ve thought about lately. |
| **Cognitive Appraisal** | ***Threat Appraisal***  *Coded where individuals showed evidence of each subcategory (e.g. perceived severity of CVD risk).* | - Perceived severity of CVD risk   The degree to which people think a particular condition is a severe risk - high/low/neutral level of severity e.g.   - “Bowel cancer is a serious illness” - “Oh 17% is not a very high number is it, so I’ll be fine” | *Low Severity*  **HP:** OK so your heart age is 68, how old are you at the moment?  **P:** 66  **HP:** 66, so it's a couple of years older.  **P:** How did you make that out?  *Neutral Severity*  **HP:** Your heart age is 60 alright, erm  **P:** But that’s what I am but I’m supposed, supposed to be better than I am is that what you’re saying?  **HP:** Yeah because you’re very slim  **P:** Yeah OK  *High Severity*  **HP:** Right on average you should expect to survive until the age of 84 without a heart attack, or a stroke.  **P:** Oh my. |
|  |  | - Consequences of CVD - Discussion about consequences of developing CVD e.g. - “I know developing CVD will reduce my quality of life” | No example available. |
|  |  | - Perceived vulnerability to future CVD/CVD-related events   Perception of personal likelihood to develop a particular condition - high/low/neutral personal relevance.   - “How likely am I to develop CVD?” - “Does that mean I’m likely to have a heart attack?” | *Low personal relevance*  **HP:** OK so your heart age is 68, how old are you at the moment?  **P:** 66  **HP:** 66, so it's a couple of years older.  **P:** How did you make that out?  **HP:** (Laugh) alright, because of the info of the stats that I have put in here.  *Neutral personal relevance*  No example available.  *High personal relevance*  No example available. |
|  |  | - Intrinsic (e.g., pleasure) and extrinsic (e.g., social approval) rewards for not addressing CVD risk. - “Being a curvaceous woman makes me happy so it doesn’t bother me that I am overweight” - “I don’t want to give up smoking because my wife smokes too, it’s something we enjoy together” | *Extrinsic rewards*    **P:** You see the thing is I’m trying to get my wife to stop.  **HP:** Oh she smokes as well?  **P:** Yeah, but she won’t, she will not and she’s got COPD.  **HP:** Oh honestly.  **P:** Yep, yep, yep.  **HP:** Gosh  **P:** Yep  **HP:** Oh yeah that’s very naughty isn’t it?  **P:** Yep |
|  |  | - Neutral response to discussion of risk   *Patient responds in a neutral manner to the risk discussion. For example, the patient could respond with ‘hmm’, ‘right’, ‘okay’. Generally very short, one word responses.*  *Not enough information is present in the patient response to classify as either positively or negatively engaged in the discussion.* | **HP:** So, this is telling me today that your heart age is 71  **P:** Right  **HP:** A lot of that is to do with your smoking.  **P:** Hm |
|  |  | - Consequences of CVD risk factors   *Split into two categories:*   - *Acceptance/awareness of health implications to engage/not engage in adaptive behaviour*   *Here the patient acknowledges or demonstrates awareness of the related health implications to engagement with an adaptive behaviour (e.g., undertaking physical activity).*  *And:*   - *Acceptance/awareness of health implications to engage/not engage related to maladaptive behaviour*   *Here the patient acknowledges or demonstrates awareness of the related health implications to engagement with a maladaptive behaviour (e.g., smoking).* | *Acceptance/awareness of health implications to engage/not engage in adaptive behaviour*  **HP:** OK and when you’re walking are you slow, steady or brisk would you say?  **P:** Brisk  **HP:** Brisk. Which is brilliant because it’s put you down into that active category  **P:** Gets the heart rate up, yeah  **HP:** Yeah gets your heart rate upwards.  *Acceptance/awareness of health implications to engage/not engage related to maladaptive behaviour*  **HP:** So it gives you food for thought.  **P:** Hmm hmm. You haven’t told me anything I didn’t already know.  **HP:** I know, I know, I know I didn’t…  **P:** So yeah |
|  |  | - Perceived severity of CVD risk factors   *Similar to perceived severity of CVD risk yet this category is related to patient-practitioner discussion of CVD risk factors specifically (e.g., cholesterol, blood pressure etc.).* | *Low Severity*  **HP:** I mean, you might find that when you are doing it at home your readings are absolutely fine.  **P:** They are what they are.  *Neutral Severity*  No example available.  *High Severity*  **HP:** Erm the other one in the red is that one cholesterol one which we were talking about there.  **P:** Oh my God that’s well up isn’t it? |
|  |  | - Clarification of results   *Related to when a patient asked questions and sought clarification about their test results from the practitioner.* | **HP:** Your HDL, this figure, that’s your good cholesterol and we want to get this one higher  **P:** Right so what is it, it’s on 1.5 at the moment?  **HP:** Yeah 1.5 which is a good figure erm…  **P:** So what what’s a good one?  **HP:** Its above 1 that is that is a good figure  **P:** Oh that’s a … yeah OK |
|  |  | - Perceived vulnerability of CVD risk factors   *Similar to perceived vulnerability to future CVD/CVD-related events* *yet this category is related to patient perceived vulnerability to related CVD risk factors specifically (e.g., cholesterol, blood pressure etc.).* | *Low personal relevance*  (in relation to smoking)  **P:** I wouldn’t have thought that it would count now.  **HP:** Well no because it seems a lot of time to regenerate.  **P:** Eighties, Nineties, 2000 – it is over thirty years ago.  *Neutral personal relevance*  No example available.  *High personal relevance*  **HP:** So let’s just have a look, we haven’t done a waist circumference it’s just… if you’d like me to do one I can do so we’ve got one for future reference?  **P:** Yeah, we can do it but I’m sure it’s too big (laughter), no that’s fine I don’t mind.  **HP:** Are you happy with, yeah yeah, I’ll em, it’s not the most flattering because it’s just below your tummy button so I’m sorry.  **P:** OK you’ve got to find it first. |
|  |  | - Perceived vulnerability to other medical conditions   *Similar to perceived vulnerability to future CVD/CVD-related events* *yet this category is related to patient perceived vulnerability to other medical conditions (e.g., cancer).* | **P:** I have gone through a bit of a stage last year thinking… I was going to die of everything you know, just eh oh god you get into that stage in life when you be oh really so. |
|  |  | - Perceived severity of other medical conditions   *Similar to perceived severity of CVD risk yet this category is related to patient perceived severity of other medical conditions (e.g., cancer). This is sometimes, but not always, in comparison to CVD.* | *Low Severity*  No example available.  *Neutral Severity*  No example available.  *High Severity*  **P:** I’m going to… my massive fear factor is cancer  **HP:** Yeah  **P:** Beyond belief.  **HP:** Because you’ve got that within the…  **P:** Mums died of it, all my aunties have died of it, my granny died on mum’s side, well in fact to be honest they all did, but of an age we lost a lot in their 60s early 60s.  **HP:** Earlies, so that’s your fear factor as well isn’t it?  **P:** That massively is yeah, yeah |
|  | ***Coping Appraisal***  *Coded where individuals showed evidence of each subcategory (e.g. Self-efficacy to engage in adaptive coping).* | - Self-efficacy to engage in adaptive coping   The belief that one is capable of performing the behaviour   - “I am confident that I can change my diet” | No example available. |
|  |  | - Response efficacy of adaptive coping   The recommended behaviour will be effective in reducing the threat   - “Changing my diet would improve my health and lower my risk of CVD” | No example available. |
|  |  | - Response cost of adaptive coping   Barriers than inhibit the performance of the adaptive behaviour   - “Healthy eating costs more money so it’s easier for me to stay as I am” | (In relation to physical activity)  **HP:** And you like physical exercise, don’t you? Do you ever cycle at all?  **P:** Yes, in the summer. I am a bit of a fair-weather cyclist, so I do yeah. |
|  |  | - Response cost of adaptive coping (previous coping)   *Similar to response cost of adaptive coping above yet this category relates to discussion of previous coping attempts, rather than current or future coping attempts.* | **P:** Well up to a couple of years ago, I was doing some mountain ones, I don’t do it on the road anymore, because of my joints and things. But I do half-marathons yes. |
|  |  | - Facilitators to adaptive coping   *This relates to practitioner-patient discussion about any factors that may positively influence or facilitate engagement with adaptive coping.* | **P:** I am a bit of a fitness …  **HP:** Are you? OK.  **P:** If I don’t get my exercise I get all sort of ratty! |
| **Coping Modes** | ***Adaptive Coping***  Coded where individuals appeared to be positively engaged with biomedical/lifestyle/psychosocial intervention discussion - apparently listening and engaged in the conversation; accepting of what is being said/suggested. | Biomedical Intervention   - GP/PN/HCA appointment referral - Medications (for blood pressure/cholesterol) - Reducing blood pressure - Cholesterol re-test - Diabetes prevention   Lifestyle Intervention   - Referral to lifestyle programmes - Weight reduction & management - Increase physical activity - Lifestyle changes - Diet related   - Increase fruit and vegetable intake   - Increase omega-3 fatty acid intake (e.g., fish)   - Decrease fat intake   - Decrease dietary cholesterol   - Decrease sugar intake   - Decrease alcohol intake   - Decrease salt intake   - Unhealthy snacking   - Portion control - Smoking cessation - Alcohol consumption   Psychosocial Intervention (E.g., referral to Healthy Mind etc.) | (In relation to weight management):  **HP:** So average survival free of heart attack or stroke is 84.1 years OK? So how do you feel about that?  **P:** Oh I will make more of an effort to lose some weight.  (In relation to reducing blood pressure):  **P:** So do I make the appointment today.  **HP:** Yes to see me in a month’s time for your erm blood pressure check  **P:** OK |
|  | ***Maladaptive Coping***  Coded where individuals appeared to be negatively engaged with biomedical/lifestyle/psychosocial intervention discussion - apparently listening and engaged in the conversation, but dismissive of what is being said/suggested (already doing all I can (e.g., already feels that they are very active and eat well); not interested in making changes suggested (e.g., like smoking; hate physical activity)  Neutral responses were coded when individuals did not appear to be engaged in biomedical/lifestyle/psychosocial intervention discussion – not engaged in the conversation (e.g., passively takes information, but no clear plans for further contact). | Biomedical Intervention   - GP/PN/HCA appointment referral - Medications (for blood pressure/cholesterol) - Reducing blood pressure - Cholesterol re-test - Diabetes prevention   Lifestyle Intervention   - Referral to lifestyle programmes - Weight reduction & management - Increase physical activity - Lifestyle changes - Diet related   - Increase fruit and vegetable intake   - Increase omega-3 fatty acid intake (e.g., fish)   - Decrease fat intake   - Decrease dietary cholesterol   - Decrease sugar intake   - Decrease alcohol intake   - Decrease salt intake   - Unhealthy snacking   - Portion control - Smoking cessation - Alcohol consumption   Psychosocial Intervention (E.g., referral to Healthy Mind etc.) | Negative Engaged:  (In relation to reducing alcohol consumption):  **P:** Pretty much says don’t drink so much doesn’t it?  **HP:** Yeah well that’s that other thing.  **P:** Yawn  **HP:** It is the drink because…  (In relation to reducing blood pressure):  **HP:** No, no. Healthy body – healthy mind, so… It is just part of your health check. Would you like to book an appointment for your blood pressure re-check? I can do it for you now or you can book at reception when it is convenient for you.  **P:** Whatever. I would leave it a month or so.  **HP:** Yes, about four weeks?  **P:** Yes. The reason being is that I will be back on my normal exercise and diet. I don’t know if that makes a blind bit of difference to blood pressure?  Neutral response  (In relation to blood pressure re-check):  **HP:** We will do another reading towards the end and if it is still above 140 on the top and 85 there,  **P:** Hm  **HP:** we will have to get you back again for another blood pressure re-check.  (In relation to smoking cessation):  **HP:** Yeah, OK. Right and on average you could expected to survive to age 79 without a heart attack or stroke but, if you gave up smoking you would increase that risk by 4.5 years OK  **P:** Hmm Hmm. |
